# Limb body wall complex in a still born fetus: a case report

**DOI:** 10.1186/1757-1626-1-86

**Published:** 2008-08-12

**Authors:** Nishad Plakkal, Joseph John, Sajini Elizabeth Jacob, J Chithira, Sowmya Sampath

**Affiliations:** 1 Department of Pediatrics, Pondicherry Institute of Medical Sciences, Kalapet, Pondicherry, 605 014, India; 2 Department of Pathology, Pondicherry Institute of Medical Sciences, Kalapet, Pondicherry, 605 014, India

## Abstract

We report the case of a male, low birth weight, stillborn fetus of 36 weeks gestation with limb body wall complex. An interesting and rare feature noted in the propositus was the absence of the left subclavian artery and complete absence of the left upper limb. These findings seem to favor the vascular theory in the pathogenesis of this condition.

## Introduction

The Limb Body Wall Complex (LBWC) is characterized by severe congenital anomalies, chief of which are thoracoschisis, abdominoschisis, limb defects, and exencephaly [1]. In our index case, apart from thoracoabdominoschisis, an interesting feature noted was an absent left subclavian artery and the complete absence of the left upper limb.

## Case report

The mother was an unbooked, 24 year old primigravida, with an uneventful antenatal period. There was no history of consanguinity or family history of any malformations. There was no history of intake of drugs other than hematinics and calcium during the pregnancy. No antenatal ultrasonogram had been done. She had been clinically diagnosed to have polyhydramnios prior to being referred to our institute. At 36 weeks of gestation, she was admitted with preterm onset of labour and delivered a stillborn fetus weighing 1900 g.

**Figure 1:**
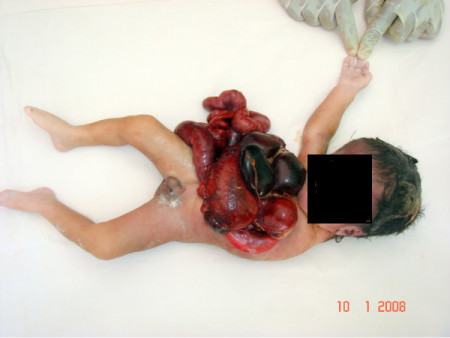
**Picture showing the entire fetus**. Notable features evident are the large defect in the body wall (thoracoabdominoschisis) with evisceration of the contents of the thoracic and abdominal cavity and the absent left upper limb.

On external examination, the placenta was normal; the umbilical cord was short; two umbilical arteries and one umbilical vein were present in the cord. The head and facies were normal. There was amelia of the left upper limb. There was a large defect in the body wall involving the thorax and the abdomen. The sternum, the anterior costal parts of the ribs on the left side and the diaphragm were absent. There was ectopia cordis, evisceration of the lungs, stomach, liver, spleen and intestines [Figure 1].

**Figure 2:**
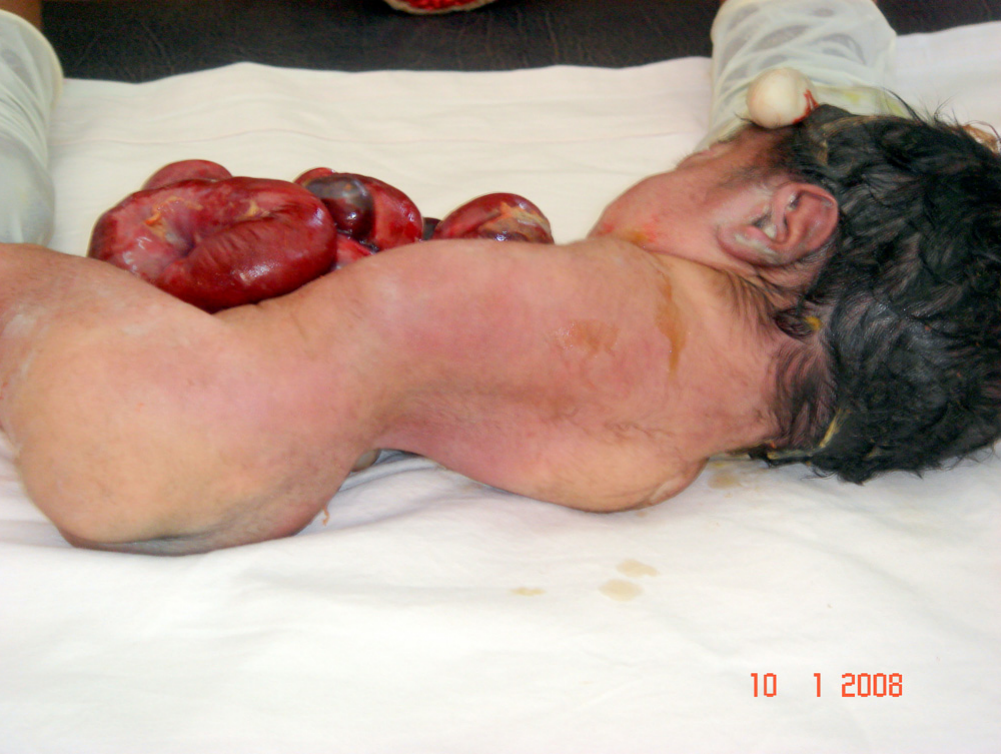
The kyphoscoliosis associated with this condition and the amelia.

At autopsy, the above mentioned findings were confirmed. Dissection of the heart revealed a patent foramen ovale. The arch of the aorta gave rise to the right brachiocephalic trunk and a small left common carotid artery. The left subclavian artery was absent. The right lung was normal, but the left was firm, hypoplastic and without lobations. The thymus was normal. The intestines were nonrotated, with the small intestine on the right and the large intestine on the left side. The small intestine was dilated and distended with thick meconium forming a cast of the intestine. The terminal ileum was stenosed. The large intestine was short, stenosed and ended in a blind pouch; the terminal part of the large intestine and the rectum were atretic; the anal canal was patent and the anal opening normal. The liver was trilobulated and the gall bladder was absent. The pancreas and spleen were normal. The kidneys and ureters were normal but the bladder was found to have a vesicourachal diverticulum arising from its apex. The external genitalia were male and both testes were intraabdominal. The cranial contents were normal. Scoliosis of the spine was present [Figure 2]. Microscopic examination of the organs was not contributory.

## Discussion

The limb body wall complex is also known as the body-stalk syndrome. It is a rare entity characterized by severe malformations. Most fetuses are aborted, either spontaneously or by medical means. Most of the remaining are stillborn. Postnatal survival for a significant duration is extremely rare, but at least one such case is reported; the child has severe physical handicaps. The etiology is unknown. No teratogen has been implicated and no genetic abnormality has been identified [2]. Familial recurrence of one sibling with LBWC and another with amniotic band sequence (ABS) [3] and another report of two siblings born with LBWC [4] have been reported suggesting the possibility of a genetic origin of the condition.

The diagnostic criteria for LBWC are still being discussed, but the most commonly quoted are those originally set forth by Van Allen et al in 1987, i.e., the presence of two of the following three malformations: (a) exencephaly/encephalocele and facial clefts; (b) thoraco and/or abdominoschisis; and (c) limb defects [1]. But these criteria imply that an infant with encephalocele with facial clefts and limb defects can be considered as having LBWC and are hence disputed, because it would be inappropriate to make the diagnosis in the absence of a body wall defect, which appears to be the primary anomaly. In 1997, Martínez-Frías suggested that those cases with body wall defect be classified in two main groups: gastroschisis, for cases with an isolated (and usually small) body wall defect; and BWC, for those cases with the body wall defect associated with other malformations, deformations, or disruptions, regardless of their clinical pattern and the possible etiology or pathogenetic mechanism. He further suggested that these two groups be separated from the ABS without body wall defects [5]. Russo et al. identified two distinct phenotypes of LBWC, one with craniofacial defects, facial clefts, amniotic adhesions and amniotic band sequences called placentocranial adhesion phenotype and the other without craniofacial defects but with imperforate anus, urogenital abnormalities, lumbosacral meningomyecoele and kyphoscoliosis called the placentoabdominal adhesion phenotype [6]. Our case resembles the placentoabdominal phenotype.

Theories on the pathogenesis of the BWC include

(a) germ disc defect with early embryonic maldevelopment [Streeter, 1930; Herva et al., 1984; Bamforth, 1992],

(b) primary rupture of the amnion leading to the formation of amniotic bands [Torpin,1965],

(c) vascular disruption [Van Allen, 1987] and

(d) disturbance of the embryonic folding process [Hartwig et al. 1989, 1991].

Though limb defects are present in the vast majority of cases, absence of a limb is seen in less than a tenth [1] and upper limb involvement is uncommon [2,5,7]. The absence of the left subclavian artery with the absence of the corresponding upper limb as in our case seems to favor the vascular disruption theory.

Antenatal diagnosis is usually made on ultrasound examination. Serum alpha fetoprotein levels are often elevated. The importance of early antenatal diagnosis in this severe condition with a poor prognosis lies in differentiating it from an isolated gastroschisis, which has a much better prognosis. Early diagnosis can be followed by medical termination of the pregnancy.

## Consent

"Written informed consent was obtained from the patient for publication of this case report and accompanying figures. A copy of the written consent is available for review by the Editor-in-Chief of this journal."

## Competing interests

The authors declare that they have no competing interests.

## Authors' contributions

NP did the literature review and prepared the manuscript, JJ made the diagnosis, critically reviewed and corrected the manuscript, SEJ conducted the autopsy, JC was involved in the literature review and manuscript preparation, SS made the final corrections, approved the manuscript and will act as guarantor. All authors read and approved the final manuscript.
